# Agent-Based Modeling Approach for Population Dynamics of the Biological Vector *Aedes Aegypti*

**DOI:** 10.1055/a-2854-0579

**Published:** 2026-04-29

**Authors:** Alexandra Schwaiger, Marco Schweitzer, Werner O. Hackl, Bernhard Pfeifer

**Affiliations:** 1 Division for Digital Health and Telemedicine, UMIT TIROL, Hall in Tirol, Austria; 2 Department of Clinical Epidemiology, Tirol Kliniken GmbH, Innsbruck, Austria

**Keywords:** cellular/hybrid automaton, agent-based models, population dynamics, *Aedes aegypti*, vector-borne diseases

## Abstract

**Background:**

The invasive mosquito *Aedes aegypti* is a major vector of arboviruses, such as Dengue, Zika, Chikungunya and Yellow Fever.

**Objective:**

As a first step toward a transmission model for vector-borne diseases, a weather-dependent population dynamic simulation for *Ae. aegypti* was developed, suitable for high weather short-term variability in the Austrian region.

**Methods:**

We developed an agent-based model, incorporating temperature- and precipitation-dependent development, mortality, movement, feeding behavior, and egg-laying. Species-specific parameters were derived from published experimental studies. Simulations were run for observed weather data from 2024 and for EURO-CORDEX climate projections (ÖKS15) for 2050 and 2080 under RCP4.5 and RCP8.5. Daily immigration of one adult female was assumed to mimic human-mediated introduction along major transport routes.

**Results:**

Under all scenarios, population development began in late May and ceased by late September. Observed 2024 conditions produced the highest population sizes (area under the curve [AUC] = 361,343 individuals), whereas all future projections resulted in substantially lower abundances, despite higher mean temperatures.

**Conclusion:**

This discrepancy was driven by stronger fluctuations in temperature and precipitation, as well as the absence of urban heat-island effects in climate projections. Under no scenario did mosquitoes survive winter, indicating that long-term establishment is highly unlikely. The model provides a foundation for future extension toward spatially explicit virus transmission simulations, and for assessing the public health implications of climate change in alpine regions.

## Introduction

Infectious diseases are a leading cause of death for children and young adults, caused by pathogens of different types, including viruses, bacteria, parasites, prions, or fungi. 1 The transmission of communicable diseases can either be direct (sexual transmission, vertical transmission from mother to child) or via a vector, such as mosquitoes or ticks. Once a vector has acquired a pathogen by one or more infectious blood meals, the pathogen can multiply within the arthropod, and transmission in subsequent meals is possible 2 for competent vectors. The vector competence is the ability of a mosquito to ingest, replicate, and transmit a virus by biting, so viral particles in saliva and replication of a virus need to be demonstrated. 3 For some diseases, also vertical transmission within the vector population is possible, in which case transmission could also take place in the first blood meal.

Environmental conditions, especially temperature and rain, are the main influencing factors for the population dynamics of mosquitoes. 4 5 6 In the Austrian federal state of Tyrol, climate change projections indicate warmer winters and altered precipitation regimes. 7 Such changes may modify habitat availability, shift species composition and increase the suitability of alpine regions for invasive mosquito species.

For Europe, flaviviruses causing Zika, Dengue, and West Nile fever, and the alphavirus causing Chikungunya were identified as potential threats in the future. 8 The most competent biological vector for transmission of flaviviruses causing Zika, Dengue, and the alphavirus causing Chikungunya is the mosquito *Aedes aegypti* . 9 10 11 *Aedes aegypti* is better adapted to warmer regions, and the risk to spread as far north as Austria is currently comparably low. Nonetheless, it is of great interest for strategic planning, for public health implications, how changing weather conditions could influence their spatial distribution.

The life cycle of most mosquitoes comprises aquatic as well as aerial stages. Summarized by Abd, 12 eggs that are below water surface can develop into larvae. Larvae of nearly all mosquito species rely on atmospheric oxygen and must periodically ascend to the water surface to breathe. The larval stage can be subdivided into four instar stages; larvae molt/shed their skin (exuviae = remaining skin) four times during development. After the last instar stage, larvae develop into pupae, where they do not feed, and emerge as adults at the end of this stage. Female adults mate once shortly after emerging and store sperm within the spermatheca to fertilize all eggs during their lifetime. To lay eggs, females of most species first need to feed on blood, in some species more than one blood meal is required. 12 The sequence of events from a blood meal to egg laying is called the gonotrophic cycle.

Aquatic stages are more vulnerable to environmental influences since they cannot move to a different habitat, and the influence of precipitation and competition is highly complex. Eggs need to be submerged under water to prompt hatching, and larval stages depend on sufficient water for survival, but if the amount of rain exceeds the capacity of habitat, larvae are being flushed out. On the other hand, after a period with increased rain, more new habitats are available, and populations of mosquitoes grow. 13

*Aedes aegypti* , also known as *Stegomyia aegypti* , displays opportunistic behavior, making the species highly successful. It preferably cohabits with humans, breeds in artificial containers, will bite day or night, and can endure low water quality. 14 In Europe, the species is currently only established in Madeira.

Due to the lack of overwintering skills in cooler regions, the distribution of *Ae. aegypti* is limited to warmer regions. Nonetheless, in the 1950's, the species was well established in all countries around the Mediterranean Sea, Black Sea, and Caspian Sea. 15 The reason why the species disappeared from Europe is unclear, possible reasons are malaria vector control measures with DDT and competition with other mosquito species.

Classical mosquito population models often rely on deterministic mathematical approaches, using ordinary differential equations (ODEs) to represent different life stages (egg, larva, pupa, adult). A prominent example is CIMSIM, developed by Focks et al. 16 This dynamic life-table model simulates daily cohort development of *Ae. aegypti* within a 1-ha area. It calculates detailed water temperatures and habitat conditions for nine container types, integrating sun exposure, filling method, evaporation, and meteorological data. Aquatic development is temperature-dependent, and mortality is modelled exponentially.

Other models use stochastic approaches. Otero et al. 17 developed a Markov-chain model with five population states (eggs, larvae, pupae, females before oviposition, females after oviposition). Ten temperature-dependent events govern stage transitions, death, and egg laying using thermodynamic relationships.

Another option are cellular automata (CA) 18 : CA are grid-based systems where each cell takes a discrete state and updates synchronously according to local rules defined by its neighborhood, with famous examples such as Conway's Game of Life showing how simple rules can generate complex dynamics. 19 20 Hybrid automata extend this idea by combining discrete modes with continuous state variables, allowing both instantaneous state changes and continuous evolution within each mode. 21

Spatial models, such as presented by Gouvêa, 22 apply CA to simulate mosquito spread across neighborhoods or larger regions. Six global parameters (birth, death, migration, sex ratio, proliferation, risk, and egg production) drive stochastic local events—egg laying, emergence, death, and movement—allowing population changes to propagate across the grid.

Agent-based approaches offer another perspective. Gunaratne et al. 23 modeled *Ae. aegypti* populations in a suburban Key West environment, with temperature-dependent duration of states and static parameters for mortality, emergence, and mating success. The model was designed to evaluate vector control strategies. The aquatic stages are combined into a single active state, whereas adults can switch among behavioral states, such as seeking food, resting, mating, and ovipositing.

## Objective

Given that many existing simulation tools are highly localized and exhibit limited transferability across geographical regions, 24 there is a clear need for region-specific modelling approaches. The mosquito model developed for Tyrol addresses this gap by incorporating local environmental conditions, thereby enabling more accurate and context-relevant predictions for regional public health decision-making. This project aims to develop a simulation tool capable of integrating projected climate data to simulate the population dynamics of *Ae. aegypti* under different climate change scenarios. Crucially, this serves as a first step toward a comprehensive transmission model for vector-borne diseases that is specifically adapted to the high, short-term weather variability characteristic of the Austrian Alpine region.

Since we plan to expand this into a spatial disease transmission model, agent-based models (ABMs) and CA are more appropriate than the classical deterministic ODE approaches or purely stochastic Markov-chain models discussed previously. ABMs simulate the system from the bottom up, allowing complex spatial distributions to emerge dynamically from local environmental rules.

## Methods

The existing agent-based simulation framework SURVIRAL was used for simulations of the population dynamics of *Ae. aegypti* . Developed by Pfeifer et al., 24 this framework began as a hybrid automaton for simulating influenza outbreaks and was later redeveloped as an agent-based framework. The underlying hybrid automaton structure, adapted from a cellular automaton for modelling infectious disease spread, comprises five core classes plus a helper class.

The simulation is based on a hybrid automaton framework comprising a spatial grid and interacting agents. Cells are defined as discrete spatial units, and the presence of agents (humans and mosquitoes) within these cells facilitates interaction dynamics.

Mosquito population dynamics are modelled through the implementation of a vector class, which encompasses life cycle stages (egg, larva, pupa, adult, dead) with sex-specific differentiation in the later stages, as well as behavioral states for female adults (e.g., blood-seeking vs. postblood meal). Transitions between these stages are governed by probabilistic rules incorporating environmental drivers, such as temperature, precipitation, flooding, and predation. Stochasticity is introduced by comparing transition probabilities with uniformly distributed random numbers. Reproducibility of the process is ensured by utilizing a fixed seed.

A species-specific subclass ( *Aegypti* ) has been defined to delineate parameterizations for development, mortality, reproduction, and behavior (e.g., flight and biting). The model parameters were derived from published literature, 13 26 27 28 29 30 31 32 33 34 35 36 37 38 39 40 41 42 43 with a particular focus on climate-dependent life cycle processes.

**Fig. 1:**
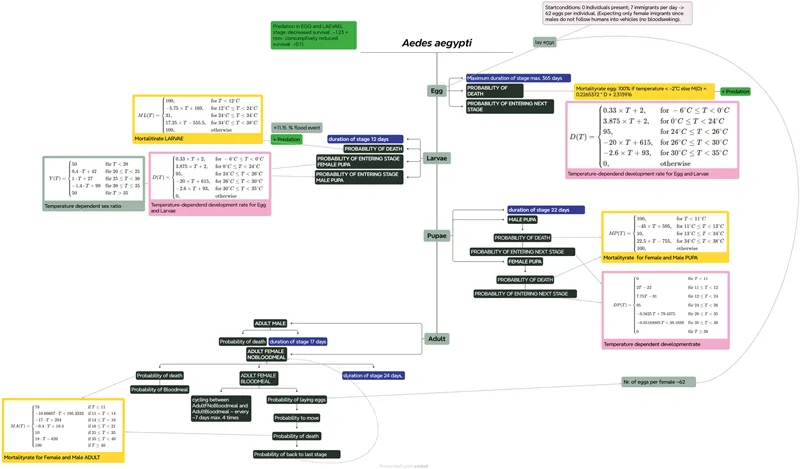
Overview of model for *Aedes aegypti* life cycle—for each agent. The four stages (gray) E = egg, L = larvae, P = pupa, A = adult; stages larvae and adult are split up into male and female states; female adults are subdivided into blood meal and no blood meal; mortality rates (yellow); duration of states (blue); development rates and sex determination (pink); sex ratio (light blue); parameters for competition, predation, and flooding (green); different options for transition (black).

For example: Survival for adult *Ae. aegypti* as determined by De Majo et al. 43 was at 12°C ∼ 22%, at 14°C ∼ 54%, at 16°C 88%, and at 20°C ∼ 90% (mortality rate was calculated 100—survival rate). Optimum survival at 21°C 35 and 38°C was identified as upper threshold for larvae by Eisen et al. 37 and 40°C for closely related species Aedes *albopictus* , 44 and it was assumed to be similar *Ae. aegypti* . To use this information for the calculation of a mortality rate MA(T) for adults a system of linear equations was created. Below 11°C, mortality was fixed at 78%. Between 11 and 14°C, mortality decreased linearly from 78 to 46%, and from 14 to 16°C it further declined to 12%. Between 16 and 21°C, mortality decreased slightly to 10%, which was maintained as a constant value up to 35°C. Above 35°C, mortality increased sharply, reaching 100% at 40°C, and remained at 100% for higher temperatures. This piecewise linear formulation reflects the reduced survival of adults at low and high temperatures and the optimal survival range at moderate temperatures. The linear equations were calculated and consolidated in the model shown in Fig. 1 .

This model integrates species-specific mortality and development functions. It also describes the maximum duration of each state, and an individual transitions to the Dead state if they exceed this maximum in their current state. Transition to the Dead state also occurs by executing the mortality rate.

For adult *Ae. aegypti* mosquitoes the flight distance on 1 day is approximately 106 m. 29 For up to 3 days, females can be blood seeking so a daily bite-rate of 0.37 is integrated. Biting does not occur below 15°C. 30 Once a female is flagged with Bloodmeal it can spend three days with moving and laying eggs (10 per day) if temperature is high enough for movement, before it falls back to no bloodmeal.

### Scenarios and Initialization of Simulation

To simulate how populations develop under changing weather conditions, it is necessary to integrate historical weather data, as well as weather data from climate prediction models. The historical data from local weather stations was obtained from GeoSphere Austria. 45

Publicly available climate projection scenarios are available through EURO-CORDEX (European branch of the Coordinated Regional Downscaling Experiment). 46 ÖKS15 provides a bias corrected EURO-CORDEX model on the GeoSphere Austria data hub. The predictions for the representative concentration pathways RCP4.5 and RCP8.5 for the years 2050 and 2080 were extracted for the coordinates nearest to the weather station in Tirol and used for simulations.

No mosquito agents are present as starting conditions. Female mosquitoes immigrate into the federal state of Tyrol via main traffic route. Only females are expected to migrate via transport, since they follow humans into vehicles while seeking for a blood meal. Eritja et al. 47 investigated how many female *Ae. albopictus* mosquitoes follow humans into vehicles and results of this study were used to estimate the number of immigrating mosquitoes for Tirol in case the species establishes in neighboring countries. Simulations were carried out with one individual immigrating per day.

## Simulation Results

Simulations were carried out on a grid of 100 × 100 cells, with each cell representing an area of 33 m × 33 m in Innsbruck. One female adult was randomly placed in the cell grid each day as an immigrant. The simulation outcomes were given a unique identification number and stored in a database.

**Fig. 2:**
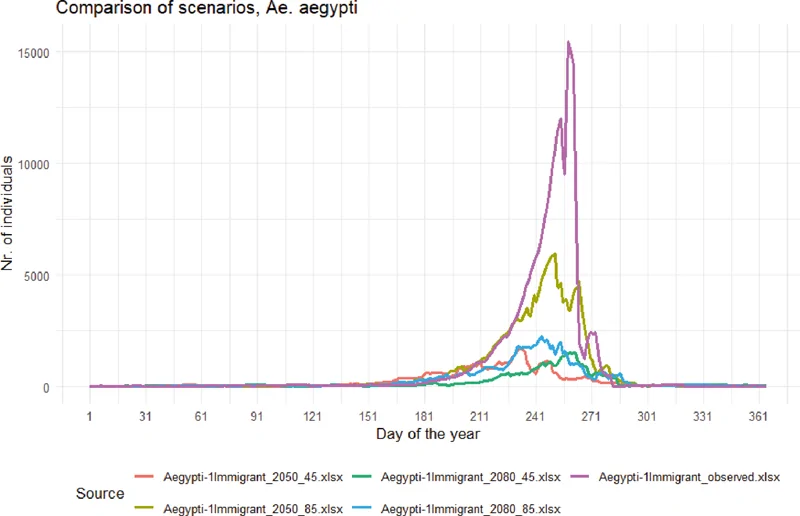
Comparison of daily total number of individuals of *Aedes aegypti* between the different scenarios.

Fig. 2 below shows the development of the total number of *Ae. aegypti* individuals under different scenarios, alongside observed weather data from 2024 and predictions for 2050 and 2080 using the RCP4.5 and RCP8.5 scenarios.

In all scenarios, the population development begins at the end of May (day 150) and the mosquito season ends around the end of September (day 270). As can be seen in Table 1 below, the comparison of the five scenarios revealed substantial differences in the overall abundance of *Ae. aegypti* individuals.

**Table 1:** Comparison of the total population of *Aedes aegypti* for all scenarios, mean, standard deviation and maximum of daily individual, area under the curve of total individuals per scenario

Scenario	Mean daily individuals	SD daily individuals	Max daily individuals	AUC total
2050 RCP4.5	245	366	1,709	89,249
2050 RCP8.5	651	1,304	5,944	237,439
2080 RCP4.5	184	321	1,523	67,167
2080 RCP8.5	312	512	2,240	113,700
Observed2024	990	2,601	15,464	361,343

Abbreviation: AUC, area under the curve.

In the simulation with observed weather data from 2024, the species reached a mean daily abundance of 990 individuals, peaking at 15,464 individuals per day and achieving a cumulative abundance of 361,343 (area under the curve [AUC]).

In contrast, the simulations with future projections showed much lower abundances of *Ae. aegypti* individuals. For 2050 under RCP4.5, the mean daily abundance decreased to 245 individuals, with a cumulative abundance of 89,249. Under the RCP8.5 scenario, this increased to 651 individuals, with a cumulative abundance of 237,439. Simulations for 2080 suggest an even stronger decline in abundances: under RCP4.5, the mean daily abundance was 184 individuals and the cumulative abundance was 67,167; under RCP8.5, the mean daily abundance was 312 individuals and the cumulative abundance was 113,700.

**Fig. 3:**
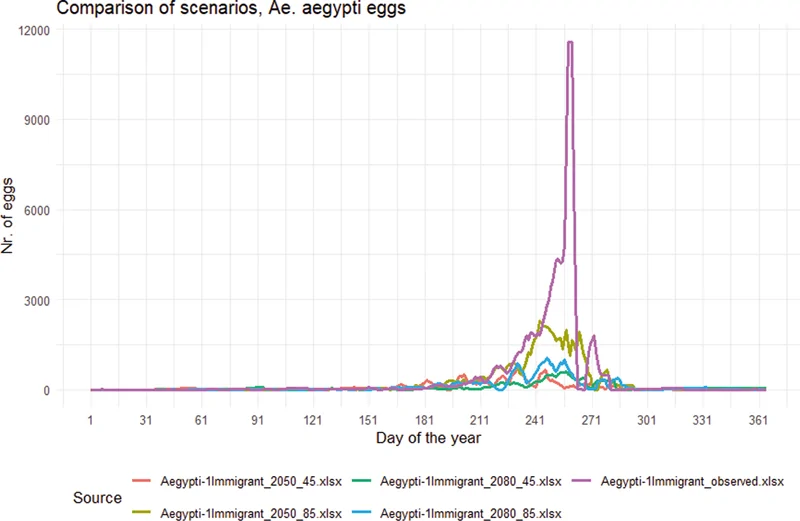
Comparison of daily total number of eggs per day of *Aedes* *aegypti* between the different scenarios.

Fig. 3 shows the development of *Ae. aegypti* egg counts under all scenarios, as well as the observed counts in 2024 and the predicted counts in 2050 and 2080 under RCP4.5 and RCP8.5.

In all scenarios, the population development begins at least 20 days later than the total individual count, with all eggs dying between the end of September and the middle of October (days 270–290). As with the individual counts, substantial differences in the overall abundance of *Ae. aegypti* eggs were revealed by the comparison of the five scenarios, summarized in Table 2 below.

**Table 2:** Comparison of *Aedes aegypti* egg abundance for all scenarios, mean, standard deviation and maximum of daily individual, area under the curve of total individuals per scenario

Scenario	Mean daily egg count	SD daily egg count	Max daily egg count	AUC total
2050 RCP4.5	86	137	738	31,314
2050 RCP8.5	238	494	2,310	87,001
2080 RCP4.5	75	123	619	27,396
2080 RCP8.5	120	212	1,061	43,754
Observed2024	424	1,380	11,582	154,691

Abbreviations: AUC, area under the curve; SD, standard deviation.

In the simulation scenario using observed weather data from 2024, the species reached a mean daily egg count of 424, peaking at 1,380, with a cumulative egg count of 154,691 (AUC). In contrast, the simulations with future projections generally showed lower egg abundances. For 2050 under RCP4.5, the mean daily egg count decreased to 86 eggs per day, resulting in a cumulative abundance of 31,314. Under RCP8.5, however, it increased to 238 eggs per day, resulting in a cumulative abundance of 87,001. Simulations for 2080 suggest a further decline under RCP4.5, with an average of 75 eggs per day and a cumulative abundance of 27,396, whereas under RCP8.5, the average daily number of eggs is 120 with a cumulative abundance of 43,754.

### Comparison of Temperatures and Rain in the Scenarios

The population dynamics of *Ae. aegypti* vary between scenarios due to differences in temperature and rainfall. The highest predicted mean daily average temperature was 14°C for 2080 under RCP8.5. The highest and lowest predicted mean daily average temperatures were 31.11°C and −9.89°C, respectively, for 2050 under RCP4.5.

In terms of daily minimum temperature, the lowest temperature predicted was −12.95°C for 2050 under RCP8.5. The highest daily maximum temperature was 39.09°C. Mean daily precipitation varies from 2.11 mm (2050, RCP4.5) to 2.78 mm (2080, RCP8.5).

While the seasonal temperature patterns are similar across all scenarios, the predicted weather patterns exhibit greater variability. Additionally, there is much stronger variation in daily maximum precipitation, with the lowest value of 36.32 mm predicted for 2050 under RCP8.5, and the highest maximum precipitation of 75.52 mm. The total amount of rain observed in 2024 was 772.7 mm. The predictions for 2080 are 1,016 mm under RCP8.5 and 821 mm under RCP4.5. The predicted data for 2050 show 901 mm under RCP8.5 and 769 mm under RCP4.5.

## Discussion

This study explores mosquito population dynamics within a modelling framework that integrates environmental drivers and species-specific processes, while explicitly accounting for uncertainty and scenario-dependent variability, given that many life cycle processes are influenced by environmental factors that are difficult to quantify and had to be estimated based on the best-available evidence.

### Weather Dependent Development and Mortality

Water availability is a key factor in egg hatching. The eggs must be submerged, but the required volume of water depends on the size of the habitat and its surface area. As both mosquito species often use artificial containers, rainfall is not the only source of water. However, no data were available for Innsbruck regarding the proportion of habitats filled by artificial sources. Therefore, a 50% reduction in egg development rate was assumed in the absence of rainfall, whereas full development was applied when precipitation exceeded 11 mm.

Once containers are filled, any additional rain will cause them to overflow, and evidence suggests that this washes out the larvae. Based on the work of Paaijmans and Nobo, 48 the effect was estimated to be 11.15% above 35 mm of rain.

The development of mosquitoes requires a certain amount of energy in the form of heat, amongst many other factors. This heat drives temperature-dependent metabolic and biochemical processes, many of which are endothermic. The degree-day model, which dates back to the early 20 ^th^ century, is frequently used in agricultural entomology and is based on this idea.

However, when temperatures are close to the thermal limits for the development and survival of the two mosquito species, the average daily temperature may lead to an underestimation or overestimation of the effects of temperature. 49 50 Jensen's inequality states that, for a nonlinear function, the mean of the function is not equal to the function of the mean. 50 As development rates are nonlinear, a day with a maximum temperature of 20°C and a minimum temperature of 10°C would not have the same effect as a day with a maximum temperature of 17°C and a minimum temperature of 13°C, even though both days have the same mean temperature. Therefore, development calculations were carried out using an effective daily temperature calculated from weighted daily minimums, maximums, and averages.

Minimum and maximum temperatures are of high importance for mortality, since mortality is 100% outside of this range. Therefore, using mean temperature alone would also be inappropriate, and minimum and maximum daily temperatures are used as a first step and compared to the species-specific cutoff temperature. Mean daily temperatures are only used if the temperature is within a viable range.

### Predation

Mortality rates for aquatic stages in literature are often derived from laboratory trials, where no predators are present. So, it was a necessary step to include the effect of predation into the models by raising mortality. But modelling the relationship between mosquito larvae and their predators in detail lies outside the scope of the current project. The effect of predation depends on a multitude of factors including, size of habitat, possible hideouts for prey, weather conditions, and predator species. For example, if water levels in a habitat are low enough, *Solenopsis invicta* (ant species is not present in Austria) can remove up to 97.2% of the eggs in a container within 24 hours. 51 The results of a meta-analysis by Russell et al. 52 of nonconsumptive reduction of survival (effect size: 0.11) and the effect of consumptive reduction (effect size: 1.23) were included. Data used in that analysis included observations from laboratory experiments and semi-field experiments. Although this provides a good approximation, the effect of predation under real-world conditions might differ significantly, especially if one considers that predator prey relationships between native predators and invasive species are largely unexamined.

### Behavioral Assumptions and Reproduction

The differentiation into male or female mosquitoes depends on genetical factors, 53 54 if there is a connection between temperature and the expression of genes determining sex in mosquitoes is not clear. To model the sex ratio as a temperature dependent function was decided based mainly on the work of Mohammed and Chadee, 31 Rozilawati et al., 55 and Delatte et al. 44 who showed that the sex ratio of *Ae. aegypti* differs with different temperatures.

*Aedes aegypti* is frequently found inside of buildings, seeking shelter in bad weather conditions or following humans for blood meals. Modelling behavior with outdoor temperatures will especially underestimate flight activity, feeding and overall survival. If temperatures drop below approximately 10°C adults will become immobile and die within a couple of days. 34 At low temperatures they will only fly short distances and neither take blood meals nor lay eggs. The presented models allow movement above 10°C and below 35°C but with a lower likelihood at suboptimal temperatures. The maximum temperature of the day was used to calculate the likelihood of the movement. Since this species will also bite outdoors during the day a short period is sufficient for consuming blood or finding breeding sites for eggs but on days with rain exciding 5 mm no flight was expected. This was the case on 28 days in observed rain in 2024, in predictions for 2025 the number of days with more than 5 mm of rain was 20 under RCP4.5 and in the predictions for 2080, 30 days under RCP4.5 scenario, and 35 days under RCP8.8 scenario.

The probability for a blood meal was set to 37% for each day which is in line with the findings of reference 33 and matches the length and number of gonotrophic cycles expected per female. Biting does not occur below 15°C 34 and is connected to moveability in the presented simulation, so calculated with maximum temperature of the day. Marini et al. 56 observed females completing the first gonotrophic cycle without taking blood (autogeny) under constant temperature regime with 15°C. This is not integrated into the model, here female adults lay eggs if conditions for blood meal where given (15°C, 37% chance per day) and temperature is within the range of flying.

### Climate Projections and Regional Context

There is a broad scientific consensus that Central Europe will experience a significant increase in temperature throughout the 21st century. Furthermore, summers will become drier, and precipitation is expected to shift to winter. 57 Several models for long-term climate predictions are available.

For this project bias corrected EURO-CORDEX models by Österreichischen Klimaszenarien ÖKS15 have been used for future projections under the RCP4.5 and RCP8.5 scenario. These projection scenarios integrate socioeconomic and technological determinants of greenhouse gas emission and other substances that influence radiative forcing. As described in the introduction RCP4.5 represents a stabilization scenario that assumes steadily declining emissions from 2012 onward. To meet this goal, a shift toward renewable energy and the deployment of CO _2_ capture and storage technologies is necessary. 58 RCP8.5 is a high greenhouse gas scenario with a modest rate of technological change and a high-energy demand on long term in absence of climate change policies. 59

Concerning the observed weather data from 2024, a comparison of mean temperatures in Tirol showed that the mean temperature in 2024 (12.5°C) was 0.9°C above the average of the mean temperatures from 2013 to 2023 (11.6°C). This relatively small deviation indicates that the difference between the projected temperatures and the observed temperatures in 2024 is minor, and consequently, the modelled population dynamics of species in the region do not differ significantly between these years. It is important to note, however, that future climate projections often do not explicitly incorporate urban heat island effects. This might lead to underestimation of temperatures in the predictions used in the simulation. The heat isle effect would lead to locally higher temperatures in cities like Innsbruck due to the retention of heat by buildings and paved surfaces. Ward et al. 60 Therefore, while regional models provide a useful approximation of expected climatic trends, the actual conditions in urban areas may experience additional warming, which could affect the population dynamics in ways that cannot be simulated without further information.

### Results and Scenario Comparison

In all simulated scenarios a development of *Ae. aegypti* was observed and revealed clear differences in abundance between simulation scenarios. The mosquitoes initiated population development at roughly the same time, between late May (day 150) and mid of July (day 200), peaking around early September (day 250). This matches the reported period for mosquito abundance in Austria. 61 However, the reproductive phase lagged compared with the initial increase in total individuals, eggs appeared later than adults.

The reproductive phase started later than the increase in total individuals, immigrated individuals can survive at lower temperatures but will not reproduce until temperatures rise. So, the timing of population onset and peak remained relatively consistent across scenarios.

A comparison of population dynamics under the predicted scenarios for 2050 and 2080 indicates that RCP.8.5 leads to warmer temperatures and more suitable rain amounts and patterns to favor population growth. Since warmer conditions favor population growth, this leads to the conclusion that temperatures in predicted date was lower or had less favorable patterns. The highest mean of daily average temperatures was 14.14°C predicted for 2080 under RCP8.5 scenario while observed in 2024 were only 12.53°C so the main reason for the lower population development will most likely be found in the pattern of temperature distribution.

Compared with the scenario based on the observed weather conditions in 2024, the populations in all the predicted scenarios did not develop as well as they did under the observed conditions. As previously mentioned, 2024 was a year of above-average temperatures, and the heat island effect was not considered in the predictions for the future climate in Innsbruck. Additionally, the stronger temperature and precipitation fluctuations in the predicted weather conditions negatively affect population dynamics, as evidenced by higher mean daily temperatures in the predicted scenarios. The influence of rain on population dynamics is less pronounced and feasible in the simulations. The number of days on which rain inhibited flight and biting ranged from 20 to 35, but development was higher after rainy periods. However, this may be offset by the effects of flooding on larvae, which are expected to be flushed out of their habitats.

### Limitations

All results presented here must be interpreted as scenario-based projections rather than deterministic predictions. The model integrates multiple stochastic processes and empirically derived parameters, many of which are uncertain, context-dependent, or only partially transferable from laboratory to field conditions. Consequently, the simulations represent plausible system trajectories under defined assumptions, but not exact future outcomes. This is a fundamental limitation of complex ecological and agent-based models: small variations in input parameters, initial conditions, or stochastic events can lead to divergent population dynamics (sensitivity to initial conditions and parameter uncertainty). From a technical perspective, simplifications were necessary, including the use of aggregated environmental inputs (e.g., daily temperature summaries), simplified representations of predation and habitat availability, and incomplete behavioral modelling (e.g., indoor resting or microclimatic effects). Consequently, these constraints may result in systematic under- or overestimation of population dynamics. In terms of public health, it is imperative that the model is utilized as a decision-support tool to identify risk windows, relative differences between scenarios, and potential system sensitivities. This approach should be distinguished from its use as a forecasting instrument, which provides exact case numbers or population sizes. Consequently, prudent planning must encompass the upper range of plausible outcomes, particularly under favorable climatic conditions conducive to vector establishment.

### Implications for Public Health

Despite these limitations, the model provides valuable insights for public health planning. Rather than serving as a predictive tool for exact population sizes, it should be interpreted as a decision-support framework to identify risk periods, compare scenarios, and assess system sensitivities.

The results highlight that local environmental conditions strongly influence mosquito dynamics, underlining the importance of region-specific models for effective surveillance and intervention strategies. Public health planning should consider a range of plausible outcomes, particularly under favorable climatic conditions that may facilitate vector establishment.

## Conclusion and Outlook

The simulations presented showed that *Ae. aegypti* would be unable to withstand winter in the federal state of Tyrol, both under the observed conditions of 2024 and under the predicted climate scenarios for 2050 and 2080. Even if female adults of this species were to immigrate daily, the establishment of populations would be highly unlikely. The observed weather conditions in 2024 resulted in higher population growth than the predicted scenarios for 2050 and 2080, despite the predicted temperatures being higher. This suggests that the temporal patterns of temperature and precipitation, as well as the absolute temperature, critically affect mosquito population dynamics. The lack of urban heat island effects in future climate projections likely contributes to the underestimation of potential population growth in urban areas such as Innsbruck. Rainfall has inhibitory as well as facilitative effects on mosquito populations, emphasizing the complex interaction between precipitation, habitat availability, and larval survival.

We established a simulation for transmission of vector-borne disease. Therefore, the model will be further developed by incorporating the heat island effect and differentiating between areas with high and low human population density. The gonotrophic cycle will also be modelled in more detail by modelling biting events rather than blood meals, since mosquitoes often bite repeatedly until they have consumed a full blood meal, especially if they are disturbed.
